# Measuring efficiency of university-industry Ph.D. projects using best worst method

**DOI:** 10.1007/s11192-016-2121-0

**Published:** 2016-09-17

**Authors:** Negin Salimi, Jafar Rezaei

**Affiliations:** Faculty of Technology, Policy and Management, Delft University of Technology, Jaffalaan 5, 2628 BX Delft, The Netherlands

**Keywords:** University-industry collaboration, Collaborative Ph.D. project, Efficiency, Best worst method (BWM), Multi-criteria decision-making

## Abstract

A collaborative Ph.D. project, carried out by a doctoral candidate, is a type of collaboration between university and industry. Due to the importance of such projects, researchers have considered different ways to evaluate the success, with a focus on the outputs of these projects. However, what has been neglected is the other side of the coin—the inputs. The main aim of this study is to incorporate both the inputs and outputs of these projects into a more meaningful measure called efficiency. A ratio of the weighted sum of outputs over the weighted sum of inputs identifies the efficiency of a Ph.D. project. The weights of the inputs and outputs can be identified using a multi-criteria decision-making (MCDM) method. Data on inputs and outputs are collected from 51 Ph.D. candidates who graduated from Eindhoven University of Technology. The weights are identified using a new MCDM method called Best Worst Method (BWM). Because there may be differences in the opinion of Ph.D. candidates and supervisors on weighing the inputs and outputs, data for BWM are collected from both groups. It is interesting to see that there are differences in the level of efficiency from the two perspectives, because of the weight differences. Moreover, a comparison between the efficiency scores of these projects and their success scores reveals differences that may have significant implications. A sensitivity analysis divulges the most contributing inputs and outputs.

## Introduction

In existing literature, collaboration is viewed as a way to increase the innovation potential and capacity of firms, which are the key factors of constant competition in a world with a high rate of technological change (Faems et al. 2005). Collaboration with universities, among others (e.g. competitors, suppliers, customers, government laboratories), enhances a firm’s innovative ability (Pavitt 2003). Several benefits are derived from the collaboration between university and industry on innovation and the economic performance of the partners (Lööf and Broström 2008). University-industry collaborations are beneficial to the two partners (Lebeau et al. 2008; Azagra-Caro et al. 2010), as they can access resources, skills, data (Albors 2002) and human capital (Lin and Bozeman 2006). By considering the benefits of collaboration for both university and industry, their relationship can take on various forms, such as scientific publications, patents, joint R&D projects, consultancy and attending conferences and meetings (Bekkers and Bodas Freitas 2008).

The importance and benefits of more collaborative forms compared to formal and non-collaborative forms of working together between university and industry have been emphasized in literature (D’Este and Patel 2007; Perkmann et al. 2013; Salimi et al. 2015a). There are some evidences that, if both university and industry take part in the collaboration actively, the success rate increases (see Salimi et al. 2015a, 2016 for a detailed discussion).

A Ph.D. project, carried out by a doctoral candidate, is one interesting way for university and industry to work together. Ph.D. projects can be done not only in the form of a collaboration between university and industry but also in a non-collaborative form (where the university is the only organization involved). Salimi et al. (2016) call the former type of Ph.D. ‘collaborative Ph.D. project’, and the latter ‘non-collaborative Ph.D. project’.

The value of Ph.D. graduates in university-industry collaboration has been highlighted in some studies (Stephan et al. 2004; Cruz-Castro and Sanz-Menendez 2005; Thune 2009, 2010). Doctoral candidates are not only seen as a channel of knowledge transferring among university and industry, they are also source of knowledge creation and they can maintain the connection between university and industry (Thune 2009).

With regard to Ph.D. collaborations, the focus in existing literature has been exclusively on the evaluation of the outputs (i.e. the evaluation of collaboration success) (see Butcher and Jeffrey 2007; Salimi et al. 2015b) or the investigation of factors that cause research projects to fail (see Kelly et al. 2002), with far less attention being paid to the inputs, while one of the challenging issues in organizing the research and training program of Ph.D. candidates is how to increase the quality, efficiency and relevance of the doctoral education by taking both the inputs and the outputs of collaboration into account. There are different programs that financially support doctoral candidates who work on projects between university and industry, such as Industrial CASE studentships in UK and the Industrial Ph.D. programs in Norway and Denmark (European Commission 2003). However, the efficiency of Ph.D. projects is by no means guaranteed.

Economists state that a producing unit is ‘technically inefficient’ if it is possible to produce more outputs with the current level of inputs or, equivalently, it is possible to produce the same outputs with fewer inputs (Thursby and Kemp 2002). By borrowing this concept, and seeing a Ph.D. project as a production unit, one could say that a Ph.D. project may be inefficient if it uses more resources while yielding similar academic outputs. Efficiency is a concept that should be considered differently from success, as, for instance, two projects can be categorized as being successful (because they both have similar outputs), while one of them may be more efficient because it uses less inputs. To be more precise, we name one project efficient because of its higher outputs levels while using the same levels of inputs or producing the same outputs while using less inputs.

In the context of university-industry relationships, the efficiency of university technology transfer has been studied by Anderson et al. (2007), while the efficiency of university intellectual property licensing has been examined by Thursby and Kemp (2002). However, the efficiency of some channels of university-industry collaboration, such as collaborative Ph.D. projects, has yet to be studied. This study takes the first step.

As such, the first aim of this study is to consider the inputs and outputs simultaneously to assess the efficiency of the collaboration. We aim to measure the academic and industrial outputs of collaborative Ph.D. candidates based on: the number of publications and citations received by publications (academic outputs) and the number of patents and received citations by patents (industrial outputs). While these are considered to be the ‘outputs’ of the Ph.D. project, they also require ‘inputs’. In fact, collaboration is inherently a costly and risky activity and to realize outputs, things like supervision are crucial in research collaboration (Thune 2009). To measure efficiency, we use the ratio of outputs over inputs. To identify the importance (weight) of different inputs and outputs, we use a recently developed multi-criteria decision-making method [Best Worst Method (Rezaei 2015, 2016)]. In addition to its scientific contribution, this study has several practical implications for research project partners to measure and improve efficiency.

It is important to note that all these identified outputs and inputs may not have the same importance from the point of view of different collaboration partners. In other words, efficiency may be evaluated differently by each partner, as each of them has their own goals and reasons to be involved in the collaboration. The way that the efficiency of university-industry collaboration is measured depends on how it is defined and evaluated (Butcher and Jeffrey 2007). To increase the efficiency of a project, it is important to know how partners evaluate the outputs and inputs. As such, the second aim of this study is to examine how different partners in a collaborative Ph.D. project evaluate efficiency differently.

The remainder of this paper is organized as follows. In “Efficiency in collaborative Ph.D. projects” section, we review existing literature on the academic outputs of collaborative Ph.D. projects and on the required inputs, as well as literature on measuring the efficiency of collaboration. In “Measuring efficiency using BWM” section, we propose a methodology to identify the weights of academic outputs and required inputs for measuring the efficiency from two perspectives. In “Data collection to determine the weight of outputs and inputs” and “Data collection to measure efficiency of collaborative Ph.D. projects” sections, we present our empirical analysis and discuss the findings. The paper ends with the conclusions, implications and future research directions in “Conclusions” section.

## Efficiency in collaborative Ph.D. projects

In the context of university-industry relationships, comparing inputs and outputs is identified as a productivity evaluation tool (Anderson et al. 2007) and has been used to assess the source of growth in university licensing (see, Thursby and Thursby 2002) and the efficiency of university technology transfer (see, Thursby and Kemp 2002; Anderson et al. 2007). We simply define efficiency as “the ratio of the weighted sum of outputs over the weighted sum of inputs”. In general, there are two approaches to calculate this ratio. One approach uses data envelopment analysis (DEA) (Charnes et al. 1978; Banker et al. 1984), in which for different decision-making units (Ph.D. project in this study) we would have different weights for inputs and outputs. The second approach uses unified weights for all the decision-making units. In such cases, a multi-criteria decision-making method is usually applied to calculate the weights of the inputs and outputs. In this research, we follow the latter approach, that is to say we use BWM, which is a multi-criteria decision-making method designed to calculate the weights for the inputs and outputs in collaborative Ph.D. projects. The main motivation of following the second approach, in this paper, is to find a way to see the possible differences or similarities between the two aforementioned perspectives with respect to the importance of different inputs and outputs, which is not achievable following the first approach.

A collaborative Ph.D. project is defined as “a project with a typical duration of 3–4 years and which involves a university, a firm, and a Ph.D. candidate, all working together to meet (common or individual) expectations.” (Salimi et al. 2016).^1^ The type of partners in the collaboration (three partners), the type of knowledge (scientific knowledge) and the duration of the collaboration (typically 3 years or longer) make collaborative Ph.D. projects different from other types of collaboration (Salimi et al. 2015a).

To calculate the weights of the inputs and outputs, we first have to identify the relevant outputs and inputs in the collaborative Ph.D. project, based on its characteristics. Salimi et al. (2015b) identified four dimensions as performance of this type of collaboration: number of publications, number of patents, number of received citations by publications and number of received citations by patents. Thune (2009), after reviewing the literature on Ph.D. candidates, also found that scholarly productivity (i.e. publications and presentations), commercial productivity (i.e. patent and trade secrets) and the future career of Ph.D. candidates are outputs of collaborative Ph.D. projects.

As mentioned above, to measure efficiency, not only do we need to know the outputs, but we also need to consider the resources (inputs) used to realize the outputs.

In a collaborative Ph.D. project, supervisors play an important role in maximizing the outcome. In other words, supervisors’ role in providing a suitable learning environment (Salminen-Karlsson and Wallgren 2008) makes the supervision dimensions essential to be considered as inputs in collaborative Ph.D. projects. Some of the supervision dimensions are: the knowledge of university supervisor(s) regarding the specific topic of the Ph.D. study, the knowledge of firm supervisor(s) regarding the specific topic of Ph.D. study (Butcher and Jeffrey 2007), academic position of university supervisor(s), and the scientific degree of the firm’s supervisor(s). One other dimension that can be considered as a required input is the number of meetings between partners (Butcher and Jeffrey 2007) especially meetings between Ph.D. candidates and their supervisors. In fact firms can enhance their innovation performance through the tacit knowledge embodied in Ph.D. candidates (Mangematin 2000). This dimension includes the frequency of meetings between the Ph.D. candidate and the university supervisor(s), the frequency of meetings between the Ph.D. candidate and the firm’s supervisor(s), and the frequency of meetings between the university and firm supervisor(s). There are some other dimensions, such as the quality of communication between the collaboration partners, the level of the supervisors’ enthusiasm, and the level of the supervisors’ openness to new ideas that are needed to get benefit from the collaboration (Butcher and Jeffrey 2007).

Although participation in the collaboration voluntary (Ansell and Gash 2008), it is important to keep in mind that the different motivations the partners have for engaging in collaboration may impact their evaluation.

A collaborative Ph.D. project is an organizationally complex project since it involves three main partners (university, industry and Ph.D. candidate) (Salimi et al. 2015a). The evaluation of the outputs of the collaboration may be different for different partners, depending on their perspective (Bekkers and Bodas Freitas 2008). The evaluation of the inputs of collaboration may also be different for different partners. More precisely, because each partner engages in the collaboration based on their own motivations and goals (which can overlap), different evaluations of collaboration efficiency are to be expected.

It is important to keep in mind that different aims and motivations of the university, firm and Ph.D. candidates may affect the efficiency evaluation. From a university perspective, drivers for working together with a firm are access to research funding, which can increase their research capacity, and access to facilities and technologies, which increases speed of discovery time (Dooley and Kirk 2007). On the other hand, some of the firms’ drivers are access to scientific competencies (Dooley and Kirk 2007), research facilities, research skills and academics (Meyer-Krahmer and Schmoch 1998; Dooley and Kirk 2007; Thune 2009; Salimi and Rezaei 2015). Finally, from a Ph.D. candidate’s perspective, one reason to be involved in collaboration can be having improved career opportunities because of the experience with both industry and university. People can be involved in collaboration for external motivations, such as prestige, funding and publications, or internal (personal) motivations, such as solving interesting problems and personal compatibility (Hara et al. 2003). Generally speaking, doing Ph.D. research can be considered a job for students in a situation where employment is low (Mangematin 2000), while, in some cases, students have non-financial incentive to invest in education, and for them, taking part in a Ph.D. program can be seen as satisfying their own personal interest (Mangematin 2000).

To summarize, assigning the same weight to all academic outputs and the required resources (inputs), and investigating only from one partner’s perspective provide a limited view of the efficiency of a collaborative Ph.D. project. By knowing the weight of each dimension, it is possible to evaluate the efficiency of collaborative Ph.D. projects from the perspective of all the partners involved. Collaboration efficiency can be measured by collecting data from all the partners (industry, university, and Ph.D. candidate) in an ideal situation. However, in this study, we measure efficiency from the perspectives of university supervisors and collaborative Ph.D. candidates. Easier access to Ph.D. candidates and university supervisors, compared to industry supervisors, is relevant from a practical viewpoint, which is why the higher response rate has motivated us to evaluate efficiency from their perspective.

## Measuring efficiency using BWM

In this section, we describe the methodology we used to evaluate the efficiency of Ph.D. projects.

Consider Ph.D. project *k,* which produces *n* outputs with the values of $O_{k1} ,O_{k2} ,\, \ldots ,O_{kn}$, using *m* inputs with the values of $I_{k1} ,I_{k2} ,\, \ldots ,I_{km}$. If we consider the importance of *n* outputs and *m* inputs as $w_{1}^{O} ,w_{2}^{O} ,\, \ldots ,w_{n}^{O}$ and $w_{1}^{I} ,w_{2}^{I} ,\, \ldots ,w_{m}^{I}$, respectively, the efficiency of Ph.D. project *k*, $E_{k}$ is calculated as follows:

1 $$E_{k} = \frac{{\sum\limits_{j = 1}^{n} {w_{j}^{O} O_{kj} } }}{{\sum\limits_{i = 1}^{m} {w_{i}^{I} I_{ki} } }}$$

The inputs and outputs levels (scores) can be gathered using a questionnaire, or through observation, or secondary databases. The weights of the inputs and outputs can be identified using a multi-criteria decision-making (MCDM) method. The data for the MCDM method can be collected from the Ph.D. candidates and supervisors. There are several MCDM methods, in this paper we use a new method called best worst method (BWM) (Rezaei 2015, 2016). We selected this method mainly because (1) it uses a very structured way to gather pairwise comparison data which results in highly reliable results; (2) It uses only two vectors instead of a full pairwise comparison matrix, which makes it an excellent method when data collection is costly with respect to time and money; (3) It is easy to understand by the evaluator, and also easy to revise by the evaluator in order to enhance the consistency level of the comparisons. The BWM has been successfully applied to other problems such as logistics and supply chain management (Rezaei et al. 2015, 2016a, b), risk management (Torabi et al. 2016), and innovation management (Gupta and Barua 2016). The steps of the BWM are as follows (we describe these steps to calculate the weights for inputs, the same procedure is used to calculate the weights for outputs).

- *Step 1* Determine a set of inputs.

In this step, we identify *m* inputs $\left\{ {I_{1} ,\,I_{2} ,\, \cdots ,\,I_{m} } \right\}$ used by the Ph.D. project. This can be done based on literature review and/or expert opinion.

- *Step 2* Determine the best (e.g. most desirable, most important) and the worst (e.g. least desirable, least important) input according to the decision-maker perspective. This is only a selection, no quantitative task or comparison is done in this step.
- *Step 3* Determine the preference of the best input over all the other inputs, using a number between 1 (input *a* is equally important to input *b*), and 9 (input *a* is extremely more important than input *b*). The result is a best-to-others (BO) vector:

$A_{B} = \left( {a_{B1} ,\,a_{B2} ,\, \ldots ,\,a_{Bm} } \right)$,where $a_{Bj}$ indicates the preference of the best input *B* over input *j* and $a_{BB} = 1$.

- *Step 4* Determine the preference of all the inputs over the worst input, using a number between 1 (input *a* is equally important to input *b*), and 9 (input *a* is extremely more important than input *b*), which results in the others-to-worst (OW) vector:

$A_{W} = \left( {a_{1W} ,\,a_{2W} ,\, \ldots ,\,a_{nW} } \right)^{T}$,where $a_{jW}$ indicates the preference of the input *j* over the worst input *W* and $a_{WW} = 1$.

- *Step 5* Find the optimal weights $\left( {w_{1}^{*} ,w_{2}^{*} , \ldots ,w_{n}^{*} } \right)$.

The aim is to determine the optimal weights of the inputs, such that the maximum absolute differences $\left\{ {\,\left| {w_{B} - a_{Bj} w_{j} } \right|,\left| {w_{j} - a_{jW} w_{W} } \right|} \right\}$ for all *j* is minimized, which is translated to the following min–max model:

$$\hbox{min} \,\mathop {\hbox{max} }\limits_{j} \,\left\{ {\,\left| {w_{B} - a_{Bj} w_{j} } \right|,\left| {w_{j} - a_{jW} w_{W} } \right|} \right\}$$

$$\text{s.t.}$$

2 $$\sum\limits_{j} {w_{j} } = 1$$

$$w_{j} \ge 0,\,{\text{for}}\,{\text{all}}\,j$$

Problem (2) is transferred to the following linear problem:

$$\hbox{min} \,\xi^{L}$$

$$\text{s.t.}$$

$$\left| {w_{B} - a_{Bj} w_{j} } \right| \le \xi^{L} ,\quad{\text{for}}\,\,\,\, {\text{all}}\,\,\,\, j$$

3 $$\left| {w_{j} - a_{jW} w_{W} } \right| \le \xi^{L} ,\quad{\text{for}}\,\,\,\, {\text{all}}\,\,\,\, j$$

$$\sum\limits_{j} {w_{j} } = 1$$

$$w_{j} \ge 0,\quad{\text{for}}\,\,\,\,{\text{all}}\,\,\,\,j$$

Solving problem (3), the optimal weights $\left( {w_{1}^{*} ,w_{2}^{*} , \ldots ,w_{n}^{*} } \right)$ and $\xi^{L*}$ are obtained. $\xi^{L*}$ is considered as a consistency index. That is, the closer the value of $\xi^{L*}$ to zero, the higher the level of consistency of the comparisons.

The weights of the outputs can be identified in the same manner. Once we have the weights of the inputs and the outputs, as well as the scores for the inputs and the outputs of a Ph.D. project, we can calculate the efficiency of the project using (1).

## Data collection to determine the weight of outputs and inputs

To examine the efficiency of collaborative Ph.D. projects from the perspective of different collaboration partners, we need to collect different data. First, we collected data from Ph.D. candidates and university supervisors to determine the importance (weight) of outputs and inputs in collaborative Ph.D. projects. This data was gathered from all departments^2^ of two technical universities in the Netherlands, Eindhoven University of Technology (TU/e) and Delft University of Technology (TU Delft). We chose TU/e and TU Delft, as both have a long track record of collaboration with industry and this provides us enough data for our research. More precisely, TU/e is based in the ‘Brainport’ region, which surrounded by several high-tech firms such as Philips, ASML and NXP. TU Delft, is also in “Randstad area” which contains, for instance, Schiphol airport and the largest European seaport in Rotterdam.

We asked university supervisors (including assistant, associate and full professors) and Ph.D. candidates engaged in collaborative Ph.D. projects to fill in a questionnaire. In this step, based on BWM, we asked the respondents to evaluate the importance of different outputs and required inputs of collaborative Ph.D. projects. The outputs considered in this study are: the number of publications, patents and their received citations, while the required inputs are: academic position of the university daily supervisor, academic degree of the partner’s (firm or public research organization—PRO) daily supervisor, level of university and its partner supervisor’s knowledge regarding the Ph.D. topic, meeting frequency among Ph.D., university and its partner supervisor. We received a total of 87 complete and valid responses, which is a response rate of (87/321) 27 %.^3^ Seventy two percent of the supervisors already supervised more than one collaborative Ph.D. projects during their career. Thirty two percent of the Ph.D. candidates are in the third or final year of their Ph.D. study. More detailed data can be found in Table 1. As shown in the table, male respondents make up 81 percent of all respondents. Furthermore, 55 percent of the respondents are Dutch.

**Table 1:** Descriptive data concerning collaborative Ph.D. candidates and university supervisors

Groups	Number of respondents	Nationality: Dutch/others	Gender: male/female
Collaborative Ph.D. candidates	37	14/23	25/12
Professors (Assistant/Associate/Full)	25/7/18	34/16	45/5

### Weights of outputs and inputs

As discussed above, to measure the efficiency of collaborative Ph.D. candidates, we need to determine the weights of different outputs and inputs. Table 2, shows the importance (weights) of the inputs and outputs of collaborative Ph.D. projects from the perspective of university supervisors and collaborative Ph.D. candidates, resulting from the application of BWM.

**Table 2:** The weights of the outputs and inputs

	Weights from the perspective of university supervisor	Weights from the perspective of collaborative Ph.D. candidate
*Outputs*		
Number of publications	0.502	0.391
Number of patents	0.135	0.136
Number of received citations of publications	0.252	0.310
Number of received citations of patents	0.110	0.163
*Inputs*		
Academic position of the university daily supervisor	0.103	0.104
Academic degree of the partner daily supervisor	0.078	0.084
Level of university supervisor’s knowledge in Ph.D. topic	0.268	0.265
Level of partner supervisor’s knowledge in Ph.D. topic	0.137	0.143
Meeting frequency of Ph.D. candidate and university supervisor	0.176	0.164
Meeting frequency of Ph.D. candidate and partner supervisor	0.106	0.128
Meeting frequency of both supervisors	0.131	0.111

As can be seen from Table 2, Column 2, from the university supervisors’ perspective, the *number of publications* is the most important output in collaborative Ph.D. projects, followed by the *number of received citations of publications*. The *Number of received citations of patents* is the least important dimension. Indeed, universities are publication-oriented, which means that they are enthusiastic about publishing the research output of a joint project as widely as possible. Collaboration with industry provides this opportunity for university professors to increase their publications (Gulbrandsen and Smeby 2005; Ponomariov and Boardman 2010), as they can benefit from exchanging the complementary knowledge (Banal-Estañol et al. 2011), data and skills. Furthermore, university professors have an incentive to publish their results quickly, to increase their (citation) impact (Dasgupta and David 1994).

**Fig. 1:**
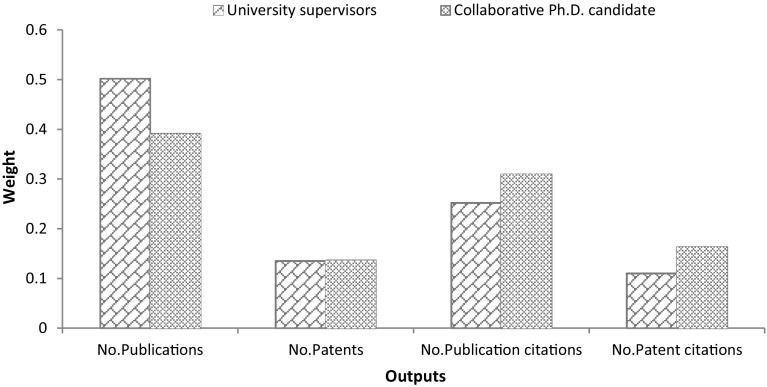
Importance of the outputs from different perspectives

The same analysis is performed to identify the outputs weights from the perspective of collaborative Ph.D. candidates (Table 2, Column 3). This result reveals a pattern similar to that of the university supervisors. However, the results show that, *number of publication* has more importance for university supervisors compared to Ph.D. candidates, while *number of received citations* of both publications and patents is more important for Ph.D. candidates compared to university supervisors. Apparently, Ph.D. candidates who opt to work in collaborative Ph.D. projects want to have more freedom in their future career, in that working in collaborative Ph.D. project facilitates not only an entry into academia but also into industry (Salimi et al. 2015b). It may therefore show that these Ph.D. candidates have an interest in not just research but also the development of their research. More precisely, Ph.D. candidates as job seekers after their graduation may need more visibility by receiving more (publication and patent) citations. Perhaps this is why the number of publications is less important to collaborative Ph.D. candidates compared to university supervisors, while the number of publication and patent citations is more important to Ph.D. candidates. More detailed information is shown in Fig. 1.

The results regarding the weights of inputs from the perspective of university supervisors (Table 2, Column 2) show that *level of university supervisor’s knowledge in Ph.D. topic* is the most important dimension, followed by *meeting frequency of Ph.D. candidate and university supervisor*. *Academic degree of the partner daily supervisor* is the least important dimension. Salminen-Karlsson and Wallgren (2008), after interviewing academic and industrial supervisors of eleven graduate students in industrial research schools, found that academic knowledge has more importance in the research projects than industrial supervisors’ knowledge. In fact, the knowledge of industry supervisors is focused more on applications and development, while that of university supervisors refers to basic academic knowledge (Salminen-Karlsson and Wallgren 2008). Our results are aligned with what Salminen-Karlsson and Wallgren (2008) found in literature. Moreover, more frequent meetings not only help create mutual trust among partners, they also provide a situation in which it is easier to transfer knowledge (Ponds et al. 2007; Bouba-Olga et al. 2012), which means that frequent face-to-face meetings between Ph.D. candidates and university supervisors, whose knowledge is a vital asset to the project, are important.

The importance of inputs from the perspective of collaborative Ph.D. candidates follows the same patterns. However, *meeting frequency of Ph.D. candidate and partner supervisor* is more important for Ph.D. candidates compared to *meeting frequency of both supervisors* (Table 2, Column 3). While university supervisors gave more weight to *meeting frequency of both supervisors* rather than *meeting frequency of Ph.D. candidate and partner supervisor.* One explanation is that Ph.D. candidates do not know much about university supervisor-firm meetings and therefore give it less importance, while university supervisors know less about Ph.D. candidate-firm meetings and therefore give it less importance.

**Fig. 2:**
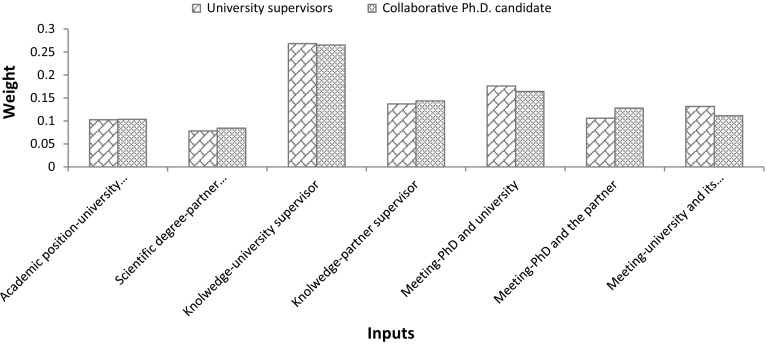
Importance of the inputs from different perspectives

As can be seen in Fig. 2, both university supervisors and collaborative Ph.D. candidates overall have very similar opinions about the importance of different inputs required for collaborative Ph.D. projects (see Fig. 2).

It is worth-mentioning that the proposed model for measuring efficiency in this study has two main features. Firstly, this proposed model is flexible. That is, not only can we enter the above inputs and outputs, but also other inputs and outputs can be considered in the model based on the problems and the cases under investigation. In this study in order to handle the problem of endogeneity due to unobservable individual characteristics, we reduced the most endogenous inputs (i.e. quality of communication among collaboration partners, level of supervisors’ enthusiasm, and level of supervisors’ openness to new ideas). Moreover, we did not consider financial aspects in our model. The reason is that in this study, we consider collaborative Ph.D. projects. This implies that these candidates receive salary from the university, and the salary scale in the Netherlands is applied to all the Dutch universities and the Ph.D. candidates consistently. In the cases that Ph.D. projects receive different salary or their projects involve some special financial aspects, this can be considered as an input, and our model is fully able to include more inputs and/or outputs.

Secondly, we define concept of efficiency as the ratio of weighted sum of outputs to weighted sum of inputs. That is, whatever is used in order to achieve the objectives of the Ph.D. project is considered as input (e.g. knowledge of supervisors and frequency of communication among partners), and whatever comes out of this project is considered as output (e.g. publications and received citations).

## Data collection to measure efficiency of collaborative Ph.D. projects

After collecting the data mentioned in “Data collection to determine the weight of outputs and inputs” section, we needed to collect data regarding collaborative Ph.D. projects, to measure their efficiency. To that end, we involved Ph.D. candidates who had completed a Ph.D. thesis as a unit of analysis.

To gather data on inputs required in collaborative Ph.D. projects, we investigated all 784 Ph.D. theses at TU/e in the years 2000–2005, from all university departments. Reading the content of the summary and preface of these theses, we identified a total of 224 collaborative Ph.D. projects. Then, after finding up-to-date contact details of the former Ph.D. candidates, we approached the full population of 224 former Ph.D. candidates. We received a total of 51 complete and valid responses, bringing our overall response rate to 23 percent. 92 percent of Ph.D. candidates had been worked on their Ph.D. 3–4 years. Only four Ph.D. candidates did not finish their Ph.D. during this period (their Ph.D. took 2, 5, 6 and 7 years respectively).This implies that our data is homogeneous in terms of the number of years that Ph.D. candidates work on their projects.

In order to check the possible existence of non-response bias, we compared differences between respondents and non-respondents, as suggested by Groves (2006). In this study through survey we got information of key demographics: nationality, gender, year of graduation, and university department. Comparing the two groups of respondents and non-respondents by *t* test showed no statistically significant differences between the two groups. So there is no strong evidence to indicate that our study suffers from non-response bias.

It is worth-mentioning here that, to carry out this step, the questionnaires were sent out to former Ph.D. candidates (224) and not to other partners in their collaboration (i.e. supervisors). One of the advantages is that the Ph.D. candidates spent a full (three to) four years on their projects and are expected to remember aspects of the collaboration very well, also because it was a one-time event for them, while their supervisors may have supervised many projects at one time and may find it harder to remember all the aspects of specific projects. Finally, tracking down a Ph.D. candidate is easier compared to finding the supervisor(s) at a university and a contact person in industry.

Regarding output dimensions, through the mentioned survey, we gathered data on the publications and patents which result directly from the Ph.D. projects of the 51 former doctoral candidates at TU/e. Moreover, to complete data on outputs of Ph.D. projects, bibliometric data was collected from the same 51 former doctoral candidates at TU/e (the respondents in the inputs survey), which included publication citation data and patent citation data from the year of publication up to eight years after that. Bibliometric data source is Elsevier’s Scopus for citation data of publications. As we mentioned, we considered all peer-reviewed papers which are determined by Ph.D. candidates (author or co-author) as resulted from their Ph.D. projects in Scopus. Scopus provides useful information including the number of publications, and details on the number of citations. Kulkarni et al. (2009) compared four databases: PubMed, Scopus, Web of Science, and Google Scholar and found that Scopus covers more peer-reviewed journals. Thomson Reuters Derwent Innovations Index (DII)/Derwent World Patents Index (DWPI) database is used for the citation data of patents. A significant advantage of this database is that it comprises patent family information, and that patent metadata has been cleaned up and harmonized. Our final dataset included a total of 132 scientific publications and 31 patents.

As we mentioned, we collected our data from only Ph.D. candidates who are expected to remember aspects of the collaboration very well. However, we conducted our survey among Ph.D. candidates who graduated between 2000 and 2005 in 2012. We are aware, though, that this issue may result in recall bias. To address this concern, we applied some remedies offered by Hassan (2006). First, we used proxy sources for reported data. In our study, all of our 4 outputs dimensions (number of publications, number of patents, citation of publications, and citation of patents) are objective dimensions and we got them from different sources. As we mentioned, in the questionnaire we asked the Ph.D. candidates to determine the name of their papers and patents which result directly from their Ph.D. projects. Then we cross-checked these papers and patents using Scopus and Thomson Reuters Derwent Innovations Index (DII)/Derwent World Patents Index (DWPI) databases respectively. In some cases we double-checked with the CVs of Ph.D. candidates to avoid both type I and type II errors as well. Moreover, we also got the information regarding citations of papers and patents from these two mentioned databases. So these outputs dimensions cannot suffer from recall bias.

Regarding inputs dimensions (academic position of the university daily supervisor, academic degree of the partner’s (firm or public research organization – PRO) daily supervisor, level of university and its partner supervisor’s knowledge regarding the Ph.D. topic, meeting frequency among Ph.D., university and its partner supervisor), we also used some remedies to decrease the effect of recall bias as much as possible. We double-checked the academic position of the university daily supervisor and academic degree of the partner’s daily supervisors through their available CV in internet and with the help of all other information that we had from the Ph.D. candidates such as the year of their graduations, department and the name of industry partners. So for these two inputs also we used other sources. Table 3 contains a description of inputs and outputs dimensions.

**Table 3:** Descriptive data concerning inputs and outputs

	Values/mean	SD	Min	Max	N
*General characteristics (through survey):*					
Nationality	Dutch: *n* = 40 (78.4 %)				51
	Others: *n* = 11 (21.6 %)				
Gender	Male: *n* = 46 (90.2 %)				51
	Female: n = 5 (9.8 %)				
Graduation year	2000: *n* = 3 (5.9 %) 2003: *n* = 9 (17.6 %)				51
	2001: *n* = 6 (11.8 %) 2004: *n* = 8 (15.7 %)				
	2002: *n* = 13 (25.5 %) 2005: *n* = 12 (23.5 %)				
University department	Applied physics: *n* = 10 (19.6 %)				51
	Biomedical engineering: *n* = 2 (3.9 %)				
	Architectural science: *n* = 2 (3.9 %)				
	Chemical engineering: *n* = 14 (27.5 %)				
	Electrical engineering: *n* = 4 (7.8 %)				
	Industrial design: *n* = 0 (0 %)				
	Industrial engineering & innovation sciences: *n* = 1 (2.0 %)				
	Mathematics & computer sciences: *n* = 8 (15.7 %)				
	Mechanical engineering: *n* = 10 (19.6 %)				
*Required inputs in collaborative Ph.D. projects (through survey):*					
Academic position of the university daily supervisor	Assistant professor: *n* = 21 (41.2 %)				51
	Associate professor: *n* = 16 (31.4 %)				
	Full professor: *n* = 14 (27.5 %)				
Academic degree of the partner daily supervisor	Bachelor or master: *n* = 8 (15.7 %)				51
	Ph.D.: *n* = 23 (45.1 %)				
	Assistant/associate/full professor: *n* = 20 (39.2 %)				
Level of university supervisor’s knowledge in Ph.D. topic	3.98	0.905	2	5	51
Level of partner supervisor’s knowledge in Ph.D. topic	3.98	0.905	1	5	51
Meeting frequency of Ph.D. candidate and university supervisor	3.16	1.614	1	5	51
Meeting frequency of Ph.D. candidate and partner supervisor	4.02	1.923	1	5	51
Meeting frequency of both supervisors	2.73	1.343	1	5	51
*Outputs in collaborative Ph.D. projects (through bibliometric):*					
Number of publi-cations	2.58	2.192	0	10	51
Total number of citations(including self-citations)	55.31	77.650	0	315	51
Number of patents	0.60	1.811	0	9	51
Total number of citations	1.27	6.190	0	43	51

To measure the level of knowledge, we asked the respondents to rate the university and its partner supervisor’s level of knowledge on the specific research topic (on a five point Likert-type scale). For frequency of meeting we considered three types of meeting: (1) meetings between Ph.D. candidates and their university supervisors; (2) meetings between Ph.D. candidates and their supervisors at the firm/PRO (partner) and (3) meetings between university supervisors and the partners. Next, the respondents were asked to rate the frequency of three types of meetings on a five-point Likert-type scale.

Another remedy to prevent recall bias is using well-structured questionnaire with very clear questions. We used this remedy for other inputs dimensions. In the questionnaire we got the required information on rating the university and its partner supervisor’s level of knowledge on the specific research topic and the meetings frequency among partners, on Likert-type scale (see Table 8 in the Appendix). One main feature of Likert scale, the linguistic qualifiers, is that it can be used to measure perceived uncertainty. Likert-type scale questions provide a situation to control the uncertainty as much as possible and prevent recall bias as well.

The third remedy, is blinding the study participants to the study hypothesis and the specific factors being studied. In our study, the respondents did not know about the main factors that we wanted to measure.

### Efficiency

As discussed in the previous section, determining the weights of inputs and outputs makes it possible to measure the efficiency of the Ph.D. candidates involved in collaborative Ph.D. projects. If we only consider the outputs of Ph.D. project, without also considering the inputs, we can measure success, which is what we find in existing literature so far. By gathering data on the inputs and outputs from 51 collaborative Ph.D. candidates, we measure efficiency from the perspective of university supervisors and collaborative Ph.D. candidates, in Columns 4 and 5 of Table 4, respectively.^4^ To show the differences between efficiency and success, we measure success by considering only the outputs of Ph.D. projects from the same two perspectives in Columns 6 and 7. Moreover, in the last column we add the unweighted success score, which is, in fact, what we usually see in existing literature.

**Table 4:** The results of efficiency measurement

Ph.D. candidate number	Grad. year	Department^a^	Efficiency-supervisor perspective	Efficiency-collaborative Ph.D. candidate perspective	Success-supervisor perspective	Success-collaborative Ph.D. candidate perspective	Unweighted success
1	2003	9	0.198	0.162	0.167	0.137	0.091
2	2001	7	0.074	0.061	0.057	0.047	0.031
3	2003	9	0.084	0.068	0.055	0.045	0.030
4	2001	2	0.480	0.467	0.410	0.391	0.283
5	2002	1	0.065	0.050	0.050	0.039	0.025
6	2002	9	0.109	0.091	0.060	0.051	0.035
7	2005	1	0.240	0.218	0.150	0.138	0.122
8	2000	1	0.233	0.210	0.203	0.182	0.127
9	2005	8	0.080	0.064	0.052	0.041	0.027
10	2004	8	0.150	0.149	0.060	0.060	0.111
11	2002	9	0.081	0.070	0.062	0.054	0.037
12	2004	8	0.000	0.000	0.000	0.000	0.000
13	2005	8	0.264	0.207	0.158	0.126	0.082
14	2004	9	0.330	0.270	0.176	0.145	0.113
15	2005	4	0.648	0.638	0.437	0.424	0.309
16	2005	8	0.353	0.274	0.204	0.160	0.103
17	2002	4	0.110	0.090	0.055	0.045	0.030
18	2003	4	0.144	0.141	0.096	0.095	0.070
19	2001	1	0.079	0.062	0.052	0.041	0.027
20	2003	2	0.367	0.349	0.259	0.250	0.182
21	2005	1	1.081	0.942	0.639	0.559	0.415
22	2003	9	0.228	0.184	0.109	0.089	0.059
23	2000	1	0.212	0.179	0.179	0.152	0.103
24	2001	5	0.488	0.551	0.296	0.338	0.525
25	2005	3	0.304	0.285	0.241	0.229	0.165
26	2001	1	0.251	0.222	0.204	0.183	0.128
27	2005	1	0.269	0.234	0.181	0.155	0.105
28	2005	9	0.213	0.172	0.162	0.131	0.086
29	2004	8	0.275	0.296	0.146	0.159	0.280
30	2004	5	0.359	0.307	0.255	0.223	0.154
31	2002	4	0.687	0.708	0.420	0.426	0.317
32	2005	4	0.468	0.460	0.296	0.295	0.219
33	2002	9	0.087	0.093	0.055	0.060	0.107
34	2002	4	0.857	0.805	0.654	0.623	0.450
35	2002	4	0.479	0.476	0.301	0.302	0.224
36	2002	3	0.083	0.071	0.059	0.050	0.034
37	2002	4	0.173	0.151	0.129	0.114	0.079
38	2005	4	0.021	0.021	0.015	0.015	0.028
39	2001	4	0.000	0.000	0.000	0.000	0.000
40	2002	8	0.241	0.196	0.167	0.138	0.092
41	2003	1	0.299	0.250	0.175	0.147	0.099
42	2004	9	0.086	0.086	0.045	0.045	0.083
43	2002	4	0.362	0.288	0.210	0.168	0.110
44	2003	9	0.546	0.496	0.408	0.367	0.256
45	2003	4	0.604	0.537	0.457	0.404	0.280
46	2002	1	0.513	0.433	0.301	0.257	0.174
47	2005	4	0.453	0.399	0.268	0.239	0.167
48	2003	5	0.452	0.357	0.258	0.204	0.132
49	2004	5	0.000	0.000	0.000	0.000	0.000
50	2004	8	0.000	0.000	0.000	0.000	0.000
51	2000	4	0.208	0.180	0.124	0.108	0.074

^a^(1) Applied Physics. (2) Chemical Engineering. (3) Electrical Engineering. (4) Mathematics & Computer Sciences. (5) Mechanical Engineering. (6) Built Environment. (7) Biomedical Engineering. (8) Industrial Design. (9) Industrial Engineering and Innovation Sciences

The results show differences in the level of efficiency and success from the two perspectives, because of the weight differences. If we examine some cases individually, we find that, for instance, Ph.D. candidates 9, 13, and 21, in Table 4, are more efficient from the perspective of their university supervisors. By examining our data, we found that these three cases have high number of publications and, because the number of publications is more important to supervisors compared to Ph.D. candidates, these cases are more efficient from the perspective of supervisors, while, for example, the Ph.D. candidates 24, 29, and 31, are more efficient from the perspective of collaborative Ph.D. candidates compared to the university supervisors. Our data shows that, in these three cases, the number of (patent or publication) citations is high compared to the number of publications. Some others, like Ph.D. candidates 38 and 42 are equally efficient with the same level from the both perspectives. In these cases, Ph.D. candidates have both publications and received citations. The Ph.D. candidates 12, 39, 49, and 50 have no outputs. When it comes to the unweighted success, we can see even more differences. For a more thorough investigation, we aim to conduct some comparison studies in the following section, to determine (1) whether efficiency is evaluated differently from the two perspectives, (2) whether success is evaluated differently from the two perspectives and, (3) whether efficiency is evaluated differently from a simple unweighted success, and (4) whether there are differences in the ranking of the Ph.D. projects with respect to the two perspectives.

### Comparison studies

#### Efficiency: university supervisors versus collaborative Ph.D. candidates

To examine the difference between the efficiency from the perspective of supervisors vs. Ph.D. candidates, we conducted the non-parametric Wilcoxon Signed Rank Test (see Table 5).

**Table 5:** Comparison results of the efficiency and success of Ph.D. candidates from different perspectives (Wilcoxon Signed Rank Test)

Ranks (Efficiency from Ph.D. candidates–Efficiency from supervisors)				Test Statistics (Efficiency from Ph.D. candidates–Efficiency from supervisors)^a^	
	*N*	Sum of ranks	Mean Rank	*Z*	−4.966^b^
Negative ranks	42	995	23.69	Asymptotic Sig. (2-sided test)	0.000
Positive ranks	4	86	21.50		
Ties	5				

Ranks (Success from Ph.D. candidates–Success from supervisors)				Test statistics (Success from Ph.D. candidates–Success from supervisors)^a^	
	*N*	Sum of ranks	Mean Rank	Z	−4.984^b^
Negative ranks	39	922	23.64	Asymptotic Sig. (2-sided test)	0.000
Positive ranks	5	68	13.60		
Ties	7				

Ranks (Efficiency from Ph.D. candidates–Unweighted success)				Test statistics (Success from Ph.D. candidates–Success from supervisors)^a^	
	*N*	Sum of Ranks	Mean Rank	Z	−5.916^b^
Negative ranks	45	1123	24.96	Asymptotic Sig. (2-sided test)	0.000
Positive ranks	2	5	2.50		
Ties	4				

Ranks (Efficiency from supervisors–Unweighted success)				Test Statistics (Success from Ph.D. candidates–Success from supervisors)^a^	
	*N*	Sum of Ranks	Mean Rank	Z	−5.820^b^
Negative ranks	43	1114	25.91	Asymptotic Sig. (2-sided test)	0.000
Positive ranks	4	14	3.50		
Ties	4				

^a^ The significance level *α* = 0.05

^b^ Based on negative ranks

The comparison results show that there is significant difference between the results of efficiency from the two perspectives. More precisely the Ph.D. projects are more efficient from the perspective of university supervisors (mean: 0.282) compared to the perspectives of collaborative Ph.D. candidates (mean: 0.255). This result can be affected by the fact that the university supervisors, who have more basic knowledge, are more publication-orientated and consequently as we found in Table 2, university supervisors attached more weight to the publication compared to the Ph.D. candidates. This result indicates the importance of considering efficiency from different perspectives as based on our results the efficiency of one specific Ph.D. project from supervisors’ perspective is generally higher than Ph.D. candidates’ perspective.

#### Success: university supervisors versus collaborative Ph.D. candidates

In order to better understanding the differences among efficiency and success from different perspectives we did the same test, this time for success from both perspectives. Conducting the non-parametric Wilcoxon Signed Rank Test to examine the difference between success from the perspective of supervisors vs. Ph.D.’s, provides results similar to efficiency (see Table 5).

The results indicate that Ph.D. projects are more successful from the perspective of supervisors (mean: 0.187) than collaborative Ph.D. candidates (mean: 0.170).

#### Efficiency versus success

As mentioned before, the existing literature only considers success (with the same weight for different item scores). As the main message of this paper is (1) considering the importance of different item scores, and (2) considering both inputs and outputs, we think that the most interesting comparison would be between the efficiency of Ph.D. projects from the two perspectives (which consider both inputs and outputs and weights for item scores) and unweighted success (which considers only outputs and ignores the importance (weight) of different item scores). The results of the non-parametric Wilcoxon Signed Rank Test for this part show a very significant difference between efficiency (from the two perspective; mean (university supervisors) = 0.282, mean (Ph.D. candidates) = 0.255) and unweighted success (mean = 0.133).

#### Rank correlation

Up to this point, all the analyses involved the level of efficiency, success and their differences from the two perspectives. It may also be interesting to examine the similarity of the orderings of efficiency and success when ranked. In literature, Kendall’s Tau and Spearman’s rank correlation coefficient are used to assess the statistical associations based on the ranks of data and it has been identified as the most popular nonparametric coefficient (Yue et al. 2002). In a situation where variables are based on a scale that is at least ordinal, Spearman’s rank correlation coefficient is suitable. Therefore, in this section, we used Kendall’s Tau, as introduced by Kendall (1955) (see Table 6).

**Table 6:** Correlation matrix using Kendall’s tau

	(1)	(2)	(3)	(4)
Efficiency from perspectives of university supervisors (1)				
Efficiency from perspectives of collaborative Ph.D. candidates (2)	0.955**			
Success from perspectives of university supervisors (3)	0.876**	0.870**		
Success from perspectives of collaborative Ph.D. candidates (4)	0.882**	0.900**	0.940**	
Unweighted success (5)	0.802**	0.837**	0.827**	0.885**

** Correlation is significant at the 0.01 level (2-tailed)

The results show that efficiency ranking from the point of view of the university supervisors is significantly correlated to the efficiency ranking from the point of view of the Ph.D. candidates. The same is true with regard to success from both perspectives. Although, there are high correlations between efficiency rankings and success rankings, these correlations are lower than the correlation between the efficiency rankings from both perspectives, and also lower than the correlation between the success rankings from both perspectives. The correlation scores between unweighted success and efficiency and success (from the two perspective), although high, are lower compared to the previous correlation scores. This result may show the importance of considering the two concepts of efficiency and success as being different from each other in a situation where the ranking of different projects is the objective. For instance, to rank the efficiency of different university collaborative projects from different departments, it is important to keep in mind that efficiency may be ranked differently than success.

### Sensitivity analysis

One of the main practical contributions of this study is to find the inputs and outputs on which a Ph.D. candidate should focus to increase his/her efficiency. These are the areas which could also result in lowering the efficiency more significantly. Although by looking at the individual inputs’ and outputs’ weights we can find which one is more important, we need to do more analysis to find the contribution of each input and output to the efficiency ratio as the inputs and outputs are all being used together in a single formula (Eq. 1). Sensitivity analysis is a scientific way to find out which inputs and outputs have more contribution to the final efficiency of a Ph.D. project. Equation (1) is used to calculate the efficiency of each Ph.D. project. Calculating the efficiency for all the Ph.D. projects we could simply find the efficiency average. Now if, each time, we increase one of the output measures by one unit for all the Ph.D. projects or decrease one of the input measures by one unit for all the Ph.D. projects and calculate the efficiency average and find the difference between the new average and the original average we could find the contribution of that output or input. Doing this for all the inputs and outputs we are able to find the most contributing inputs and outputs. Table 7 shows the results of sensitivity analysis. For instance, the first element of the table (Efficiency difference-Publication) is calculated as follows. Using the original data we calculate the efficiency score for each Ph.D. project using Eq. 1 (original efficiency score). An average of the efficiency scores can now be calculated (original average). We then add one publication to the number of publications of all the 51 Ph.D. projects. Then a new efficiency score is calculated for each project using Eq. 1. An average of the efficiency scores can now be calculated (new average). The difference between the new efficiency average and the original efficiency average is calculated as 0.054 which is shown in the table. All the other scores of the table are calculated using the same procedure, except for the inputs for which we decrease the scores by one unit.

**Table 7:** Sensitivity analysis results

	Supervisor perspective	Ph.D. perspective
*Output*		
Efficiency difference—Publication	0.054	0.040
Efficiency difference—Patent	0.019	0.019
Efficiency difference—Publication citations	0.001	0.001
Efficiency difference—Patent Citation	0.004	0.006
*Input*		
Efficiency difference—Knowledge of university supervisor	0.026	0.014
Efficiency difference—Knowledge of firm supervisor	0.016	0.012
Efficiency difference—Position of university supervisor	0.012	0.023
Efficiency difference—Academic degree of firm supervisor	0.013	0.012
Efficiency difference—Frequency of meeting between Ph.D. candidate and firm supervisor	0.006	0.006
Efficiency difference—Frequency of meeting between Ph.D. candidate and university supervisor	0.013	0.049
Efficiency difference—Frequency of meeting between university and firm supervisor	0.012	0.009

The numbers in the table show how much the efficiency average increases due to increasing an output by one unit or due to decreasing an input by one unit. The second column shows the results from the perspective of supervisor, while the third column shows the results from the perspective of Ph.D. candidates. As can been from Table 7, from among the outputs, number of publications has the most contribution followed by the number of patents from the both perspectives. The other two outputs are placed in the next places. From among the inputs, interestingly, while according to supervisors, knowledge of university supervisor has the most significant role, from the perspective of the Ph.D. candidates, this is the frequency of the meetings between the Ph.D. candidate and university supervisor which could significantly improve the efficiency. From the perspective of university supervisors the other inputs have almost the same contribution except for the frequency of meetings between the Ph.D. candidate and firm supervisor which has a much lower contribution. From the Ph.D. candidates’ perspective, position of university supervisor also has a high contribution followed by the other inputs with the frequency of meetings between university supervisor and firm supervisor and also between Ph.D. candidate and firm supervisor as the least contributing inputs. The results of the sensitivity analysis can be used to make strategies to improve efficiency.

## Conclusions

This study offers a conceptual and empirical contribution to existing literature on university-industry collaboration in two ways. Firstly, this study focuses on collaborative Ph.D. project as one type of collaborative channels between university and industry, which has thus far received little attention in literature. In existing literature, university patenting and licensing have been the main topics, rather than the “academic engagement” of universities towards industry (D’Este and Patel 2007; Perkmann et al. 2013). Secondly, in contrast to previous studies on university and industry collaboration, which only focused on success (Butcher and Jeffrey 2007; Salimi et al. 2016), this study examines a way to measure efficiency, looking at why considering different perspectives in measuring a project’s efficiency is important and why we should distinguish between the efficiency and success of projects.

This study emphasizes the importance of using different weights for the inputs and outputs of Ph.D. projects from the point of view of different partners to measure efficiency. Our results show that university supervisors evaluate inputs and outputs differently compared to Ph.D. candidates, which in turn affects their efficiency. More precisely, our results show that Ph.D. projects are more efficient from the perspective of university supervisors compared to that of collaborative Ph.D. candidates. Considering the perspectives of different partners in evaluating projects helps partners focus more on the vital aspects of projects and to improve the efficiency. Based on our results, being published is more important than any of the other outputs from the perspective of university supervisors and collaborative Ph.D. candidates. This indicates that, to be more efficient, projects should be supervised in order to produce more publications. Moreover, this result can help partners even before starting the projects. University supervisors can hire a candidate with, among other things, a better ability in writing papers. A sensitivity analysis was also conducted to find the most contributing inputs and outputs, based on which the number of publications and patens found to be the most contributing outputs, while inputs related to university supervisors (knowledge, position, meetings) are identified as the most contributing inputs.

To summarize, by following the proposed method in this study, university supervisors can measure the efficiency of their collaborative Ph.D. projects. Moreover, at a higher level, universities can also compare the efficiency of all the Ph.D. projects in one department, or even compare the efficiency of the Ph.D. projects of two or more departments, which in turn can help solve the problems involved in organizing the research and training program of Ph.D. candidates, to increase the efficiency and quality of the research projects.

The insights from our study allow us to offer suggestions for further research. To begin with, in measuring efficiency, we considered the perspectives of university supervisors and collaborative Ph.D. candidates for getting weights of inputs and outputs. For a complete picture of efficiency, we hope future studies also include the industry perspectives on weighting inputs and outputs. Moreover, we sent the survey to get data on inputs only to the former Ph.D. candidates. However, getting data from supervisors provides a better and wider view to evaluate efficiency. A further avenue of future research can be to measure the efficiency of non-collaborative Ph.D. candidates. In non-collaborative Ph.D. projects, partners (university and Ph.D. candidates) may have different reasons to be involved in the project and consequently evaluate inputs and outputs different from partners in a collaborative project. Also, comparing the efficiency between collaborative Ph.D. candidates and non-collaborative Ph.D. candidates would be another interesting avenue of future research, as would be, measuring the efficiency of other university education programs. In this study we considered the Ph.D. projects successfully defended by the Ph.D. candidates. However, considering the former Ph.D. graduates who did not complete their study (especially when such uncompleted projects result in some outputs (e.g. publications or patents)) and doing comparison study would be interesting and deserves attention as another valuable future research in this field.

As it is mentioned, the main attention of this study has been paid on evaluation of project by considering both inputs and outputs. For the case of cross-comparison among efficiency of Ph.D. projects in different faculties, citations should be normalized for differences between scientific fields. We collected our data to get the weights from two technical universities (Eindhoven University of Technology and Delft University of Technology). All the scientific fields in these two universities are engineering. This implies that our data is relatively homogeneous in terms of scientific field. Nevertheless, still there might be weights differences in different faculties of these universities. Therefore, a suggestion would be adjusting the weights by subject area. Moreover, the main feature of the proposed model in this study is its flexibility. That is, other outputs and inputs can be considered for measuring efficiency based on the field and problem under investigation. For instance, the future career of Ph.D. candidates and financial resources (in the cases that Ph.D. projects receive different salary or their projects involve some special financial aspects) are two alternative variables for outputs and inputs respectively, which can be used for measuring efficiency. The second feature is considering whatever is used in order to achieve the objectives of the Ph.D. project as input, and whatever comes out of this project as output. As such, our inputs relate to supervision characteristics and the outputs refer to publications, patents and their citations. However, one might argue that part of these inputs represents communication/interaction processes, and citations to patents and publications are impact variables. Therefore, a wider consideration (input-process-output-impact) might provide better insights. Finally, we did not take into account the inputs of the Ph.D. candidate himself/herself (e.g. how many hours (per week) a Ph.D. candidate worked on average on the project), as asking this from the Ph.D. candidate in our survey is not reliable. In an ideal situation, gathering data on inputs related to the Ph.D. candidates from supervisors is more reliable and recommended.

## Appendix

See the Table 8.

**Table 8:** Description of required inputs in collaborative Ph.D. projects

Required inputs in collaborative Ph.D. projects	Name of variable	Reference question in the questionnaire
Supervision	Academic position of the university daily supervisor	What was the position of your university daily supervisor when you started your Ph.D. project?
		Assistant professor
		Associate professor
		Professor
	Academic degree of the collaborating partner supervisor	Scientific degree of your (main) supervisor at the collaborating partner when you started your project.
		Bachelor or Master
		Ph.D.
		Professor
	Level of university supervisor’s knowledge in Ph.D. topic	How would you rate the knowledge of your university supervisor(s) in the specific topic of your Ph.D. study?
		(Scale: very low to very high)
	Level of collaborating partner supervisor’s knowledge in Ph.D. topic	How would you rate the knowledge of your supervisor(s) at the collaborating partner in the specific topic of your Ph.D. study?
		(Scale: very low to very high)
Communication	Meeting frequency of Ph.D. candidate and university supervisor	Please indicate the average frequency of supervision meetings you had with (any of) your university supervisors. (Note: this is about supervision meetings, not about other events at which you met these persons)
		More than once a week
		About every week
		About every 2 weeks
		About every month
		About every 3 months
		About every 6 months
		Less than every 6 months
	Meeting frequency of Ph.D. candidate and collaborating partner supervisor	Please indicate the average frequency of supervision meetings you had with (any of) the supervisors at the collaborating partner (Note: this is about supervision meetings, not about other events at which you met these persons)
		More than once a week
		About every week
		About every two weeks
		About every month
		About every 3 months
		About every 6 months
		Less than every 6 months
	Meeting frequency of both supervisors	What was the frequency of meetings where both the supervisors of the university and the supervisors at the collaborating partner were present?
		About every month
		About every 3 months
		About every 6 months
		About every year
		Less than every year

## Footnotes

1 In the Netherlands, compared to the other countries, the characteristics of Public Research Organizations (PROs) are to some extent similar to the firms. Therefore, PRO can be a partner of university as well as a firm in the collaborative Ph.D. projects. For more details we refer to (Salimi et al. 2015a).

2 All departments in Eindhoven University of Technology are: Applied Physics, Chemical Engineering, Electrical Engineering, Mathematics & Computer Sciences, Mechanical Engineering, Built Environment, Biomedical Engineering, Industrial Design, Industrial Engineering and Innovation Sciences. All departments in Delft University of Technology are: Architecture and the Built Environment, Civil Engineering and Geosciences, Electrical Engineering, Mathematics and Computer Science, Industrial Design Engineering, Aerospace engineering, Technology, Policy and Management, Applied Sciences, and Mechanical, Maritime and Materials Engineering.

3 Data acquisition took place between February and May 2014.

4 Moving beyond the mere considering mentioned seven required inputs and four outputs as efficiency dimensions, we look at correlation between other dimensions which may have impact on efficiency. These dimensions are: the number of years a Ph.D. candidate spent on the project (input), if the Ph.D. candidate is the first author in publications (input), and if any job was offered from university or its collaborating partner (firm/PRO) to the former Ph.D. candidate after graduation (output). We found that there is no significant correlation between mentioned dimensions and efficiency from neither the perspective of university supervisors nor the perspective of Ph.D. candidates.
